# Geochemical Characterization of Kupferschiefer in Terms of Hydrocarbon Generation Potential and Hydrogen Content

**DOI:** 10.3390/molecules30193886

**Published:** 2025-09-25

**Authors:** Irena Matyasik, Małgorzata Kania, Małgorzata Labus, Agnieszka Wciślak-Oleszycka

**Affiliations:** 1 Oil and Gas Institute—National Research Institute, 25A Lubicz Str., 31-503 Krakow, Poland; matyasik@inig.pl (I.M.); kaniam@inig.pl (M.K.); agnieszka.wcislak@inig.pl (A.W.-O.); 2 Institute of Applied Geology, Silesian University of Technology, 2 Akademicka Str., 44-100 Gliwice, Poland

**Keywords:** Kupferschiefer, generation potential, kerogen, isotopes, hydrogen, kinetics

## Abstract

The Permian Kupferschiefer shale, a key stratigraphic unit within the Zechstein sequence of the Fore-Sudetic Monocline, represents both a metal-rich lithofacies and a potential source rock for hydrocarbon generation. This study presents a comprehensive geochemical characterization of selected Kupferschiefer samples obtained from the Legnica–Głogów Copper District (LGOM) and exploratory boreholes. Analytical methods included Rock-Eval pyrolysis, Py-GC/FID, elemental analysis, TG-FTIR, biomarker profiling, and stable carbon isotope measurements. Results indicate that the shales contain significant amounts of Type II and mixed Type II/III kerogen, derived primarily from marine organic matter with minor terrestrial input. The organic matter maturity, expressed by T_max_, places most samples within the oil window. Rock-Eval S2 values exceed 60 mg HC/g rock in some samples, confirming excellent generative potential. Py-GC/FID data further support high hydrocarbon yields, particularly in samples from the CG-4 borehole and LGOM mines. The thermal decomposition of kerogen reveals multiple degradation phases, with evolved gas analysis identifying sulfur-containing compounds and hydrocarbons indicative of sapropelic origin. Isotopic compositions of bitumen and kerogen suggest syngenetic relationships and marine depositional settings, with samples from a North Poland borehole showing isotopic enrichment consistent with post-depositional oxidation. Kinetic parameters calculated using the Kissinger–Akahira–Sunose method demonstrate variable activation energies (107–341 kJ/mol), correlating with differences in organic matter composition and mineral matrix. The observed variability in geochemical properties highlights both regional and facies-dependent influences on the shale’s generative capacity. The study concludes that the Kupferschiefer in southwestern and northern Poland exhibits substantial hydrocarbon generation potential. This potential has been previously underestimated due to the unit’s thinness, but localized zones with high TOC, favorable kerogen type, and low activation energy could be viable exploration targets for natural gas.

## 1. Introduction

The Kupferschiefer (copper-bearing shales) are part of the Lower Zechstein copper-bearing formations within the Fore-Sudetic Monocline. They constitute an integral component of the copper-bearing series exploited by KGHM Polska Miedź S.A. (Lubin, Poland) in the Legnica–Głogów Copper District (LGOM). This segment of the Fore-Sudetic Monocline represents a portion of one of the largest polymetallic copper ore deposits in Europe. Copper-rich sandstone sequences were first encountered in the 1950s [1,2]. The exploitation of the deposit discovered at that time continue to this day, and the resource base is still being expanded with new, increasingly deeper seams of the Zechstein copper-bearing series [3,4]. Currently, copper ore extraction by KGHM Polska Miedź S.A. is conducted through deep underground mining in three mining facilities: “Lubin”, “Rudna”, and “Polkowice-Sieroszowice”.

Copper mineralization is primarily associated with the boundary strata between the Rotliegend (Pcs) and the first Zechstein cyclothem (PZ1), known as the Werra Cyclothem. The spatial position of the copper ore deposit is typically defined by the horizon of the Kupferschiefer; in the absence of this unit, it is delineated by the contact zone between white sandstones and dolomites. Consequently, the copper-bearing series includes the uppermost layers of the Rotliegend (Pcs), white Rotliegend sandstones (Bs1), the basal limestone (Ca0), also referred to as the boundary dolomite, the Kupferschiefer shales (T1)—comprising bituminous, clayey, and dolomitic varieties—and carbonate rocks of the Zechstein limestone (Ca1), represented by clayey dolomites, banded dolomites, and calcareous dolomites.

The formations constituting the Kupferschiefer horizon (with an average thickness ranging from 30 to 60 cm) exhibit significant variability in terms of the proportions of primary mineral components. These include clay minerals (e.g., illite, montmorillonite, chlorite), carbonates (dolomite, calcite), metal sulfides, detrital material (most commonly quartz, but also feldspars, muscovite, rock fragments, and individual grains of heavy minerals), and organic matter [5,6,7,8,9,10,11]. Variations in the proportions of these constituents allow for the distinction of several lithological variants of the shale [11,12,13]: bituminous shale, clay shale, and dolomitic shale. According to other authors [14], the following types are distinguished: black or dark gray clayey-bituminous shales, clayey-dolomitic shales, dolomitic shales, and marl shales.

Chemically, the Kupferschiefer is dominated by silica, which is the main component of the clay minerals. Carbonates are present in variable amounts. Sulfur played a significant role in ore formation processes, occurring in the shale as both sulfides and sulfates. Iron, in both its ferrous and ferric forms, is primarily found in clay minerals and as an admixture in carbonates [15].

Fine-grained, low-permeability sedimentary rocks containing organic matter have the potential to act as both source and reservoir rocks for hydrocarbons, depending on their organic matter content and thermal maturity. These rocks can also be a potential source of hydrogen [16]. Abu-Mahfouz et al. [17] state that source rocks, especially those characterized by the presence of type II-S kerogen, are known for their significant capacity to generate hydrocarbons and other hydrogen-rich gases.

Numerous petrological studies on the organic matter in the Kupferschiefer shale have shown that the dominant group of macerals in the dispersed organic matter is the liptinite group, with admixtures of vitrinite and inertinite macerals [18,19,20]. The bulk of the organic substance is of sapropelic origin, derived primarily from marine organisms. Organic matter, serving as an energy source for bacterial sulfate reduction processes, is recognized as one of the key factors contributing to the formation of Cu-Ag sulfide mineralization [21,22,23,24]. As a result of this process, ore minerals commonly co-occur with dispersed organic matter, which negatively affects ore beneficiation and copper recovery.

Despite exhibiting certain properties characteristic of source rocks, the Kupferschiefer shale is not widely considered a potential source rock for hydrocarbon generation [24,25,26]. Geochemical studies conducted in Poland within the Fore-Sudetic Monocline, as well as in the Rhine Valley in Germany and in Norway, have shown that this horizon primarily contains oil-prone Type II kerogen, with total organic carbon (TOC) values reaching up to 15.89 wt% [25,27,28,29,30,31,32,33]. Hydrocarbon generation in the Kupferschiefer source rock began between the Middle Triassic and Late Jurassic, with a peak during the Jurassic period. Unfortunately, the extremely low thickness of this shale—ranging from 0.1 to 1.3 m (average ~0.35 m)—significantly reduces its importance as a source rock on a regional scale.

Preliminary experimental results from Kupferschiefer shale samples, based on a range of geochemical analyses—including molecular composition of desorbed gases from core degassing and pyrolytic investigations using Rock-Eval and Py-GC/FID techniques—conducted by Kania [26] and Kania et al. [34], have indicated high, and in some cases even excellent, hydrocarbon generation potential. Moreover, in light of numerous reports in the literature [25,28,35,36,37], which emphasize that natural gas within the Fore-Sudetic Monocline may at least partially originate from the organic matter in the Kupferschiefer, we believe that the source rock potential in this region warrants detailed investigation and characterization.

The Permian Kupferschiefer in Poland presents an excellent opportunity to test this hypothesis, particularly in the case of immature or marginally mature shales enriched in both transition metals and organic matter.

**Figure 1:**
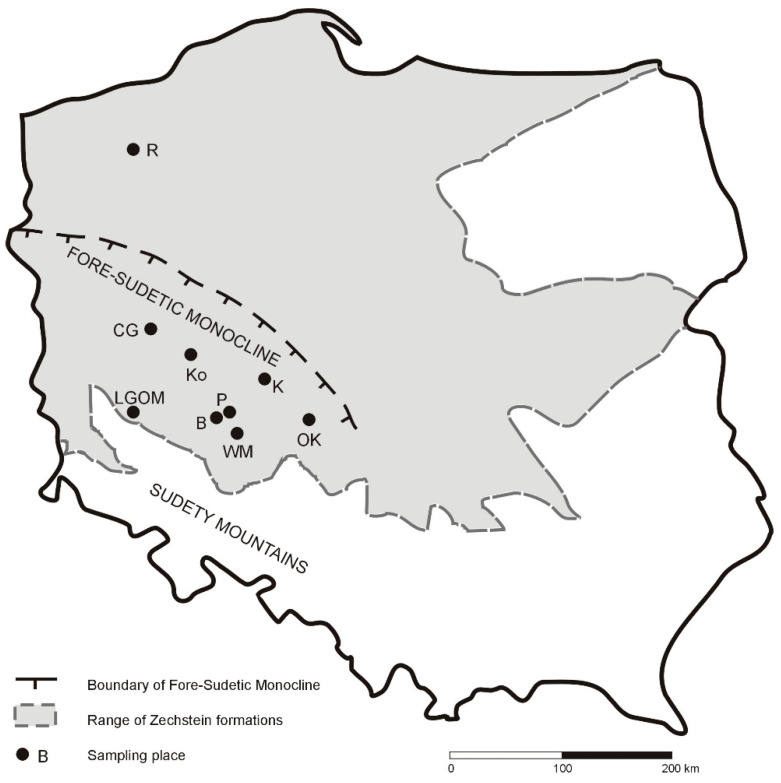
Map of Poland with sampling places (symbols as in Table 1).

The samples were collected from underground workings of a mining facility within the Legnica–Głogów Copper District (LGOM), operated by KGHM Polska Miedź S.A., as well as from several boreholes located within the Fore-Sudetic Monocline (Table 1, Figure 1). Following preliminary analyses of 38 rock samples, 10 Kupferschiefer shale samples were selected for detailed investigation. These samples are highlighted in gray in Table 1. The selection criterion for detailed analysis was based on parameters obtained from Rock-Eval pyrolysis. For each borehole, the aim was to choose the most representative samples, which simultaneously exhibited high TOC values and strong hydrocarbon generation potential.

In addition, kerogen was isolated from selected samples to determine its elemental composition, including atomic H/C and O/C ratios. For the same samples, extractable organic matter (EOM) was also obtained and subsequently analyzed to assess its genetic origin.

## 2. Results

### 2.1. Rock Eval Pyrolysis Test Results

**Figure 2:**
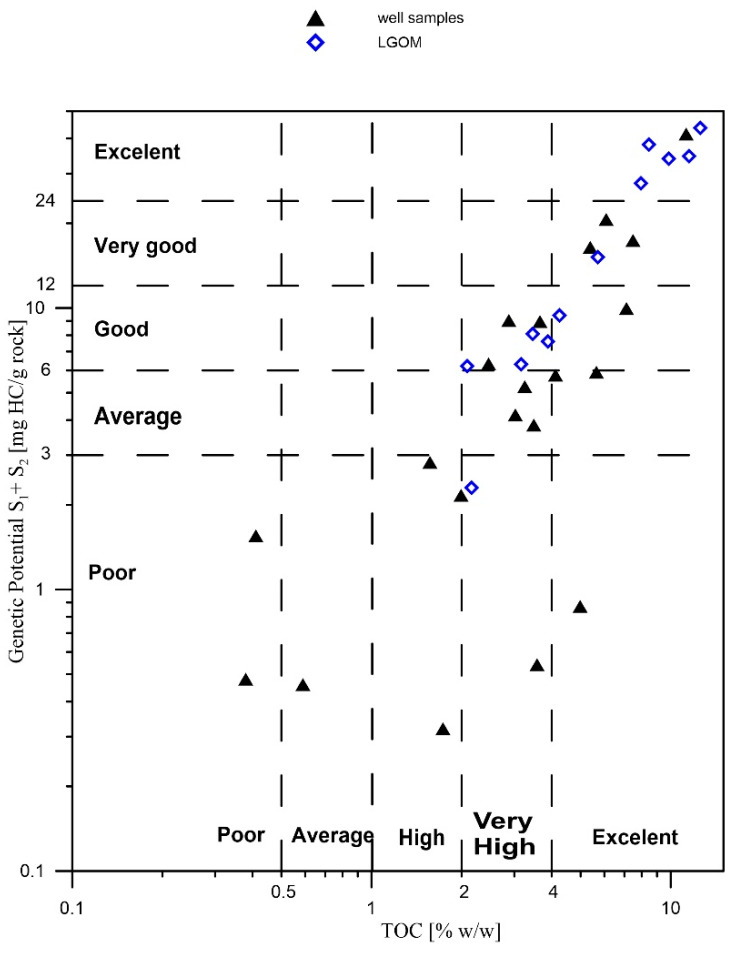
Pyrolysis S1 + S2 versus total organic carbon (TOC) plot showing generative source rock potential for the Kupferschiefer.

The results of the Rock-Eval (RE) pyrolysis studies are presented in Table 1 and Figure 2. The total organic carbon (TOC) content in the majority of the analyzed samples exceeds 2% (except for four samples), which qualifies them as very good source rocks for hydrocarbon generation. The samples richest in organic matter are Ne25-1, CG-4, and OK-2.

**Figure 3:**
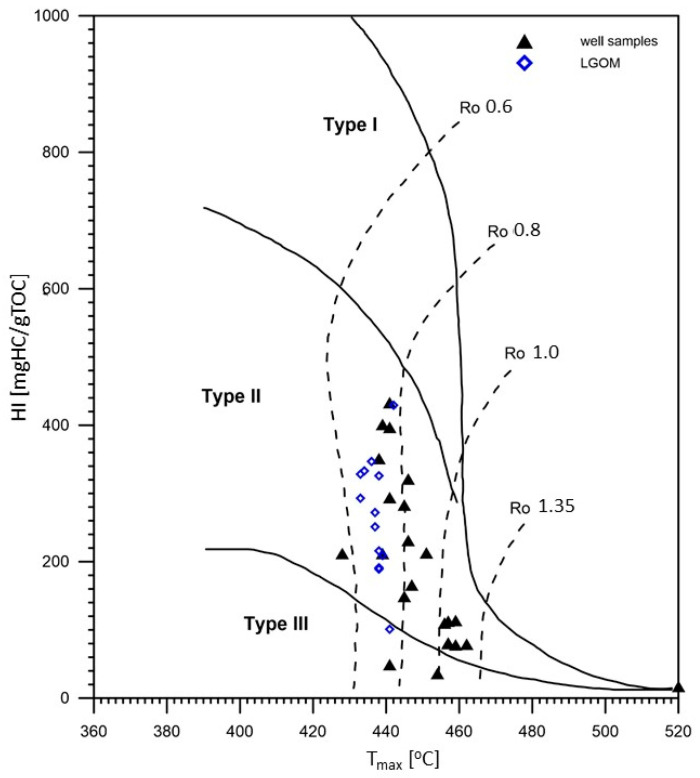
Kerogen type and thermal maturation of Kupferschiefer source rocks.

The hydrogen index (HI) values in the analyzed samples are highly variable, ranging from very low (14 mg HC/g TOC) in thermally mature samples to high (433 mg HC/g TOC) in samples with lower thermal maturity, within the range of 0.6–0.8% VRo. Based on classification diagrams (Figure 3), it was determined that the analyzed samples are dominated by Type II and mixed Type III/II kerogen, indicating a mixed origin of the source organic matter.

The oxygen index (OI) values in the analyzed samples range from 3 to 51 mg CO_2_/g TOC (Table 1). These are relatively low values, which, when considered alongside the hydrogen index, indicate a high hydrocarbon potential of the studied copper-bearing shales. The maturity level of the organic matter, expressed by the Tmax parameter, is also variable—from 428 to 538 °C—suggesting a wide range of thermal alterations, from the initial phase of thermocatalytic processes (oil window) to post-mature stages (over-mature). The S2 parameter (the amount of hydrocarbons generated during kerogen cracking in the temperature range of 300–650 °C, expressed in mg HC/g rock) is generally high—reaching over 63 mg HC/g rock in the case of sample CG-4—which confirms excellent hydrocarbon generation potential. Samples originating from the LGOM region (Lubin-Głogów Copper District) are characterized by higher generative potential parameters and lower oxidation indices, as well as a stable level of thermal maturity within the oil window (Table 1, Figure 2 and Figure 3).

Much greater differences were observed in samples from exploration wells, where the highest maturity and at the same time the lowest generation potential were found in two samples: WM-1 and K-1. This may be related to the oxidation of organic matter in the shallowest water levels of the Zechstein Sea. Low values of hydrogen potential may come from zones in which type II kerogen secondary oxidation or type III kerogen occurred.

### 2.2. Results of High-Temperature Pyrolysis Coupled with Capillary Gas Chromatography (Py-GC/FID)

Based on the obtained Py-GC/FID records, the measured quantities of pyrolysis products (at a given temperature) were grouped into three fractions, with their content normalized to 100%:

- Light hydrocarbon fraction: C_1_–C_9_;
- Liquid hydrocarbon fraction: C_10_–C_15_;
- Heavy hydrocarbon fraction: above C_15_^+^.

**Figure 4:**
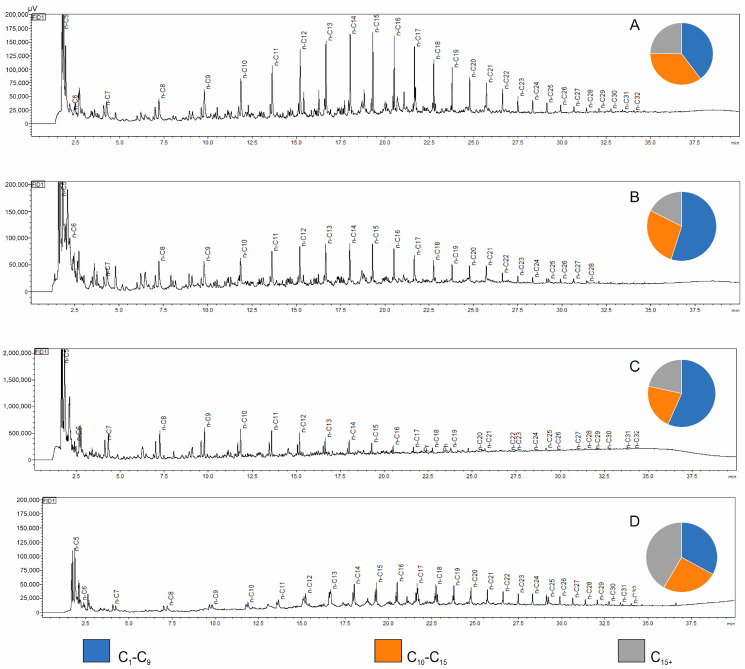
Chromatograms of pyrolysis products obtained for the samples: (**A**)—B-3, (**B**)—R-4, (**C**)—CG-4, (**D**)—Jm16-1.

The results of the fractional composition of simulated hydrocarbon generation products for the analyzed Kupferschiefer samples are presented in Table 2. Representative chromatograms from the Py-GC/FID analysis of the studied samples, illustrating the variability in composition and distribution of pyrolysis products at the set temperature, are shown in Figure 4.

Analysis of the chromatograms confirms the oil–gas character of the kerogen present in the studied rock samples. The highest Py-GC/FID yield indices were recorded for sample CG-4, where the yield exceeded 170. High Py-GC/FID yield indices, calculated per 1 mg of rock sample (although significantly lower than in the case of sample CG-4), were also observed for selected samples from the LGOM area (samples Jm02-1 and Ne25-1) and for the sample from a borehole located in the Fore-Sudetic Monocline (OK-2), which confirms their high hydrocarbon generation potential. All copper-bearing shale samples from the boreholes showed a dominant presence of light hydrocarbons in the pyrolysis products, accounting for over 70% of the total fractional composition (Figure 4B,C). The copper-bearing shale samples from the deep mining area (LGOM) are characterized by higher contents of high-molecular-weight hydrocarbon fractions, which is clearly reflected in the chromatographic profile of sample Jm16-1 (Figure 4D). For this sample, the overall pyrolysis hydrocarbon yield index was low, amounting to 8.64.

### 2.3. Occluded Gas Composition

The molecular composition analysis results for residual gas determinations were recalculated so that oxygen, nitrogen, and carbon dioxide (related to air contamination of the samples) were subtracted from the gas composition. Therefore, only the “excess” amounts of compounds separated from the rock sample remained in the gas composition. The molecular composition of the residual gas was determined in % mol/mol and then, after taking into account the container volume and the mass of the rock sample, expressed in microliters of separated gas component per kilogram of rock sample (µL/kg of rock).

Gases from the degassing of Kupferschiefer samples from underground workings of the LGOM mines were determined for the content of hydrocarbons, excess nitrogen, sulfur compounds, as well as He and H2. Detailed results of these studies are included in monograph book by Kania [26]. In this study, the content of hydrocarbons and hydrogen will be addressed only due to their potential for hydrocarbons generation.

Compared to other lithologies representing the rock formations available in the LGOM mines (sandstone, dolomite, anhydrite, and rock salt), the highest average values of desorbed gas were found for Kupferschiefer samples [26]. Throughout the entire studied profile, the highest desorbed gas content was found in the Kupferschiefer series (45%), followed by: sandstone (22%), rock salt (22%), dolomite (9%), and anhydrite (only 2%) [26]. The primary components of gas desorbed from the copper-bearing shale were light hydrocarbons and methane. The hydrogen content in the desorbed gas ranged from 0 to 158.4 μL/kg of rock for the copper-bearing shale samples. In the case of residual gas, the H2 content reached 473.5, with an average value of 160.3 μL/kg of rock.

The average composition of the desorbed gas for the tested copper-bearing shale samples indicates a dominant component of excess nitrogen at 99.17%. The remaining components include light hydrocarbons (from C1 to C10) at 0.45%, with an addition of carbon dioxide at 0.35%, as well as hydrogen and carbon monoxide at 0.01%. Similarly, the residual gas composition is dominated by excess nitrogen at 95.97%. The proportion of light hydrocarbons (from C1 to C10) is 2.07%, and hydrogen: 0.10%.

**Figure 5:**
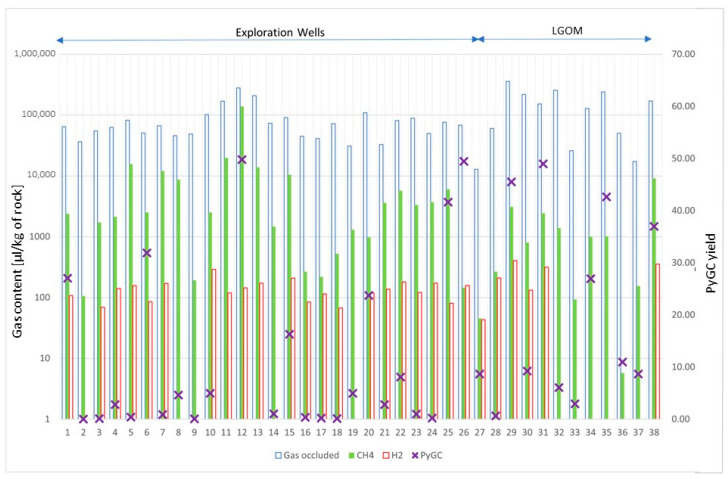
Occluded gas content.

As many authors have pointed out, hydrogen in fine-grained rock samples, which are often the source rocks for hydrocarbons, may originate from the decomposition of organic matter present in the shale. For example, Isotopic studies indicate, that hydrogen present in Kupferschiefer in eastern Germany originates from the release of hydrogen from hydrocarbons as a result of their oxidation. To confirm one of the hypotheses about hydrogen generation from organic matter in this study, open pyrolysis experiments (PyGC) were performed on Kupferschiefer samples from both the LGOM region and from exploration wells. For all samples, the total yield of produced hydrocarbons and the light fraction in the C1-C9 range were determined. Figure 5 shows the content of selected gases in all (38) samples. Due to lack of space, the samples are numbered from 1 to 38, and their assigned names are given in the Table A1 in the Appendix A. The ordinate axis on the left is scaled in units of gas volume per unit sample mass (μL/kg of rock). The scale is logarithmic because the amount of methane and hydrogen gases is relatively small compared to the total amount of gas obtained from degassing (gas occluded). On the right axis is a dimensionless yield indicator (calculated from Py-GC/FID analysis based on peak area per 1 mg of rock sample). This was used to determine the product-generating capacity of Kupferschiefer samples.

The total amount of gases released from the pore space (occluded gases) is highest for the Kupferschiefer samples from the LGOM area, exceeding 100,000 mL/g of rock, and is consistent with the hydrocarbon yield from pyrolysis of these samples. This high yield of hydrocarbon products is associated with a high hydrocarbon index (HI) and the level of thermal alteration corresponding to the main phase of the oil window (Table 1). However, when comparing the composition of occluded gas, it should be noted that the samples from LGOM mines have a lower methane content than the samples from boreholes, with comparable total amounts of desorbed gas. The hydrogen content in the desorbed gas from all samples ranges from 0 to 403 μL/kg of rock. Samples in which hydrogen is absent in the desorbed gas come from the NE and E parts of the LGOM area; However, it is difficult to find any correlation with other research results. For samples from drill holes, the lack of hydrogen can be associated with low TOC content and the lowest hydrocarbon index obtained from pyrolysis of these samples.

### 2.4. Extraction Results

Bitumens—organic compounds dispersed in rock that are soluble in organic solvents—are products of the metamorphosis of the original, petroleum-generating organic matter. The content of bitumen extracts in the analyzed samples of copper-bearing shales was highly variable, ranging from 296 ppm (sample K-1) to 6533 ppm (sample CG-4), indicating the highest presence of liquid hydrocarbon accumulation in the latter (Table 1). The nature of these hydrocarbons suggests they were generated in situ from the copper shale horizon, as evidenced by the bituminosity index (ESO/TOC) being below 100 mg/g TOC. In the studied samples, this index ranges from 14.13 to 75.19 mg/g TOC, with the lowest value observed in sample R-6, which shows a low saturation level and high thermal maturity.

The bituminous material, like the composition of pyrolysis products, exhibits significant variability, with the ratio of hydrocarbons to resin-asphaltene compounds being lower in samples from LGOM and three borehole samples (B-3, P-2, and Ko-2). Higher hydrocarbon content was found in samples that also showed high amounts of free hydrocarbons during pyrolysis, indicating accumulation of generated hydrocarbons (samples R-4 and OK-2). Furthermore, the samples Ne25-1, CG-4, and OK-2 showed the highest content of aromatic hydrocarbons (38.42%, 41.70%, and 41.10%, respectively), which may suggest a significant input of terrestrial organic matter in the source material. These same samples also show the highest HC/HZ ratios, which may further indicate the presence of weakly oxidized zones with low content of resin-asphaltene compounds.

Such zones were also discussed by Sawłowicz [38] and Puttman et al. [39] in reference to copper-bearing shales in Germany. Organic matter from oxidized zones typically contains more oxygen- and sulfur-containing compounds and is depleted in saturated hydrocarbons. This depletion of the saturated hydrocarbon fraction in bitumens was also observed in samples from the “Konrad” copper mine (not existing now) [38].

### 2.5. Isotopic Composition of Carbon

The carbon stable isotope composition was analyzed in the bitumen extract, its individual fractions, as well as in the kerogen isolated from the studied samples (Table 3).

Additionally, kerogen from sample Jm02-1 was analyzed, for which isotopic analysis of the bitumen extract and individual hydrocarbon fractions was not performed.

The results of carbon isotope composition determinations are expressed as:

δ^13^C = [(^13^C/^12^C _sample_ − ^13^C/^12^C_PDB_)/(^13^C/^12^C_PDB_)] × 1000‰ (1)

The reference value (PDB—Pee Dee Belemnite) is the carbon isotope composition of calcium carbonate from the rostrum of *Belemnitella americana* from the Peedee Formation in South Carolina (USA). The analysis results represent the difference between the isotope composition of the sample and that of PDB, expressed in per mil (‰) [40,41,42].

The kerogen samples show very similar values of stable carbon isotope composition, correlating with both the asphaltene fraction and bitumen extract, which supports previous conclusions about the syngenetic origin of the rocks and their bitumen content. Among the analyzed samples of the Kupferschiefer, sample R-4 clearly stands out, as its aromatic fraction, total extract, and saturated fraction exhibit higher contents of ^13^C.

The carbon isotope composition of kerogen isolated from the shale divides the samples into two groups: one with values around −26.0 to −26.8‰, and another with values from −27.2 to −27.5‰. The lower values correspond closely to the Cu-bearing facies on the Fore-Sudetic Monocline and the SO facies of copper shale from the North Sea [43], as well as to marine Permian-age samples. On the other hand, the higher (heavier) isotope values resemble those of Zn- and Pb-bearing facies in Germany [44], though the values still differ, possibly indicating different oxidation histories in these two basins.

### 2.6. Elemental Composition

The contents of elements C, H, N, S, O were determined from the bituminous extract obtained from selected samples of tested shales by comparing the atomic ratios (Table 4).

The carbon content in the analyzed samples of bitumen extract ranges from 79.38% for sample Jm16-1 to 87.68% for sample OK-2. Sample Jm16-1 exhibits the highest sulfur content (8.69%) among the analyzed bitumen extracts. The sulfur content in the remaining extracted bitumen samples varies between 1.03% and 4.62%. Elemental analysis of the bitumens reveals variability in the atomic H/C ratio, ranging from 0.09 to 1.47, the O/C ratio ranging from 0.01 to 0.09, and the N/C ratio ranging from 0.010 to 0.012.

**Figure 6:**
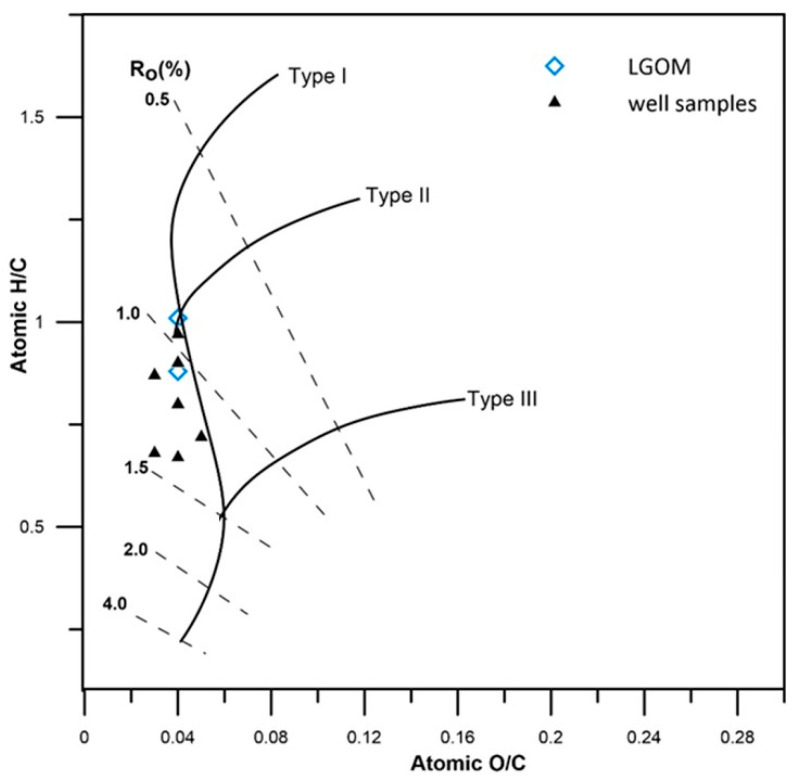
Atomic H/C to O/C ratio for kerogen from tested samples. The position of the genetic fields represents natural maturation trends of different types of kerogens as given by Hunt [46].

The results of the elemental analysis of kerogen from the analyzed shale rock (Kupferschiefer) are presented in Table 5. Correlation of the atomic H/C and O/C ratios indicates the presence of both type II and type III kerogen in the analyzed copper shales (Figure 6). The H/C ratio ranges from 0.67 to 1.01, which is consistent with values reported in the literature [25,38,45].

The highest C_ker_ content (Table 5) is observed in kerogen isolated from shale samples CG-4 and OK-2. The greatest amount of mineral matter was found in kerogen from the LGOM samples (Jm02-1 and Jm16-1). In terms of elemental composition, the lowest hydrogen content occurs in kerogen samples R-4 and R-6, which may indicate secondary oxidation during epigenetic mineralization or a significant contribution of type III kerogen [25,47]. It should also be noted that samples R-4 and R-6 are characterized by a high degree of thermal maturity.

**Figure 7:**
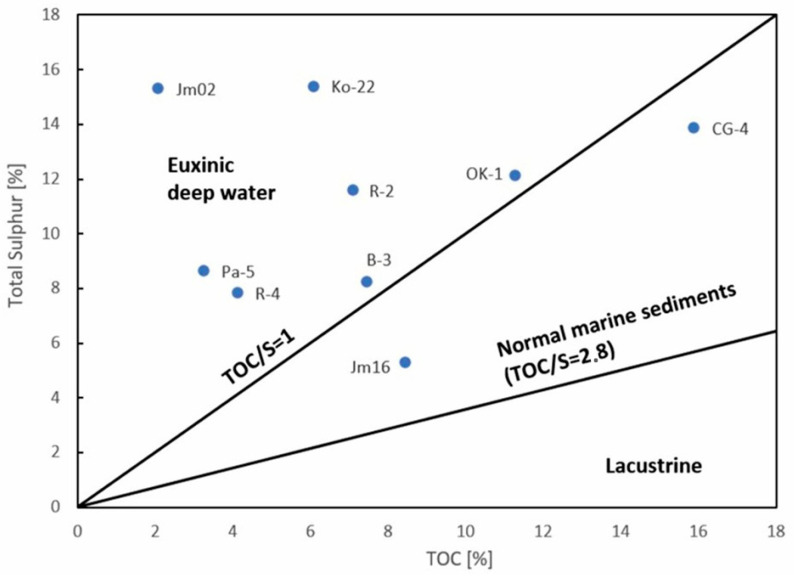
TOC (%) vs. TS (%) diagram according to Berner [48] classifying sediments according to the depositional environment.

The C/N ratio in kerogen ranges from 32.9 to 53.2, which is typical for sapropelic-type organic matter [38,45]. This type of organic matter, deposited under specific environmental conditions, is further supported by the correlation between sulfur content and TOC (Figure 7), where the majority of samples are located within the field characteristic of a low-oxygen, deep-water environment conducive to the preservation of organic matter and the occurrence of metal sulfides.

### 2.7. Genetic Characteristics of Bituminous Substance

The distribution of n-alkanes and isoprenoids indicates the presence of type II kerogen and/or mixed type II/III kerogen, exhibiting varying degrees of thermal maturity, predominantly corresponding to the main phase of thermocatalytic processes (oil window).

The genetic studies of organic matter contained in the copper-bearing shale from both the LGOM region and individual boreholes aimed to determine the sedimentary conditions of these deposits and their direct relationship to hydrocarbon generation potential, as well as to compare the genetic characteristics of samples from different locations.

**Figure 8:**
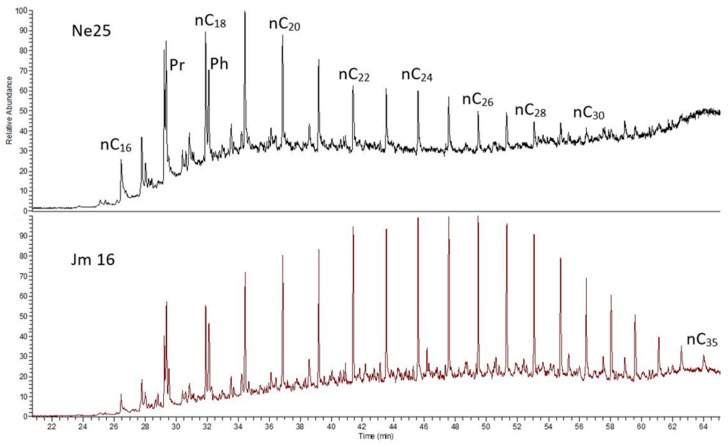
Chromatograms of n-alkanes and isoprenoids of the extract from Kupferschiefer from the LGOM mine (sample Ne25-1 and Jm16-1).

In the saturated hydrocarbon fraction, n-alkanes predominate (Table 6, Figure 8). Their distribution varies and is shifted toward lower molecular weight homologs in samples with a lower content of saturated hydrocarbons relative to aromatics, which may suggest a higher contribution of algal organic matter. Similar distributions are observed in samples B-3, CG-1, and OK-2, where the n-alkane maximum occurs around n-C20. This shift toward dominance of lower carbon number compounds (around C18) in the Kupferschiefer is attributed to an increased influx of algal material during shale deposition.

The CPI (Carbon Preference Index) values of n-alkanes are generally close to 1.0, which reflects the advanced stage of organic matter transformation. The isoprenoid structures present in the samples are represented by phytane (C20, Ph) and pristane (C19, Pr), which are products derived from phytol, the side chain of chlorophyll structures. Additionally, farnesane (C15, Fa) is also present and may originate from various primary sources. One such source is farnesol, an isoprenoid side chain found in bacteriochlorophyll structures of photosynthetic bacteria belonging to the Chlorobiaceae family.

Among the analyzed shale samples, the sample Jm16-1 from the LGOM region is distinguished by its n-alkane composition, with the distribution notably shifted toward higher molecular weight n-alkanes, extending up to n-C35 (Figure 8). According to the commonly applied interpretation of the Pr/Ph (pristane/phytane) ratio, low-oxygen conditions during early diagenesis can be inferred [49]. It is suggested that pristane is a degradation product of phytol derived from chlorophyll formed under relatively oxidizing conditions, whereas phytane is the dominant degradation product under relatively reducing conditions. Based on this interpretation, Large and Gize [50] investigated the pristane/phytane ratio in several sections of copper-bearing shale from the “Rudna” and “Lubin” mines, identifying a correlation with ore mineralization. In three sections from the “Rudna” mine, an increase in the Pr/Ph ratio was documented in the transition from copper-rich zones to overlying lead-rich zones. This may indicate an early diagenetic oxidative event or that Zn-Pb precipitation occurred under more oxidizing conditions compared to Cu precipitation.

In the present study, samples from the LGOM region exhibit Pr/Ph ratios generally close to unity (1.0 to 1.19), whereas slightly lower values were recorded for samples from exploration boreholes (ranging from 0.54 to 1.12) (Table 6).

**Figure 9:**
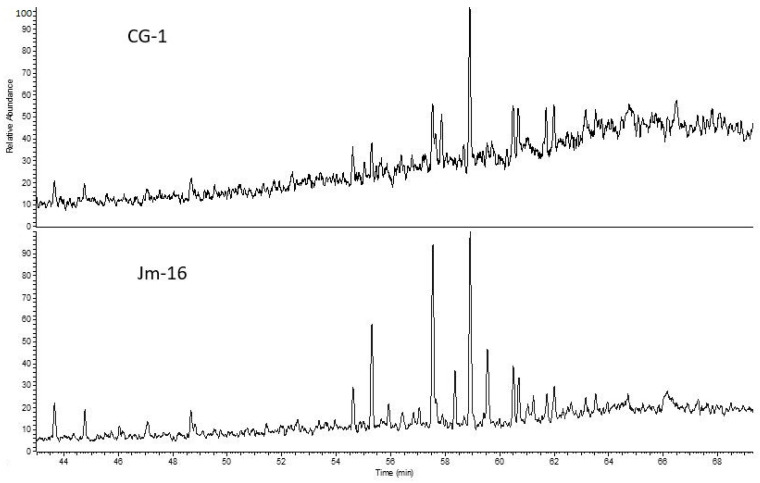
Mass chromatogram of m/z 191 saturated hydrocarbons of the sample from the CG-4 well and from LGOM (Jm16-1).

Attention was also given to the presence and composition of other biomarkers, including those from the terpane and sterane groups. Among the terpanes, hopanes and tricyclic terpanes were present, although in very low concentrations. In borehole R (sample R-2), located in the northernmost zone of the study area, no biomarkers were detected in the m/z 191 mass chromatogram, likely due to high thermal maturity. In samples from the copper mines (LGOM), biomarkers from the hopane group and tricyclic terpanes are present. These samples also contain significantly higher amounts of tricyclic terpanes and C29 norhopane, particularly in sample Jm16-1, which exhibits a lower generative potential (Figure 9). Among the borehole samples, the highest concentration of tricyclic terpanes was observed in sample P-2, which is characterized by low generative potential, elevated oxidation indicators, and shallow burial depth of the sediments.

A wide range of aromatic compounds from the phenanthrene group, as well as sulfur-containing compounds, were identified in the copper-bearing shale samples. The presence of aromatic compounds is explained by the formation of sulfides and their derivatives, which led to an increase in phenanthrene and naphthalene concentrations and a decrease in alkylated phenanthrenes and alkylated naphthalenes [29]. The analyzed samples exhibit varying trends, as the parameters derived from the aromatic compound composition are influenced not only by ore formation but also by other factors such as the circulation of formation waters.

**Figure 10:**
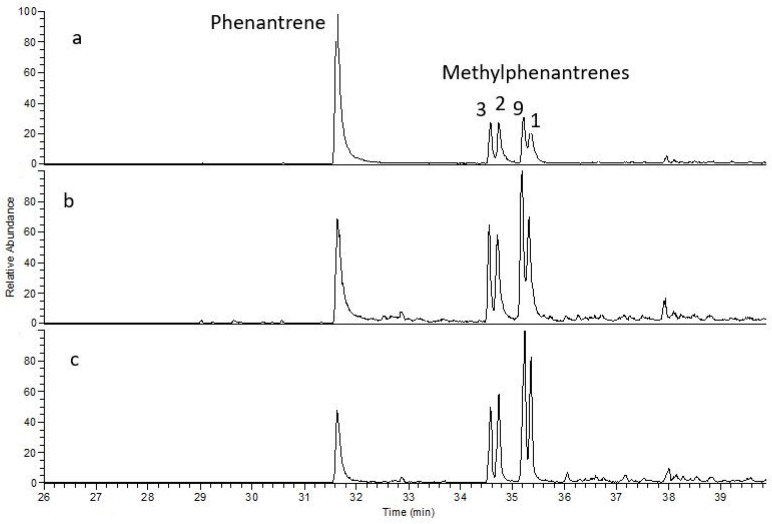
Phenanthrene and naphthalene compounds in samples from copper shale: (**a**)—Jm16-1; (**b**)—B-3; (**c**)—Ko-2. (1, 2, 3, 9—methylphenanthrene).

Sulfur-containing compounds from the benzothiophene group were present in all samples, as also noted by Rospondek et al. [51]. Among the methylated derivatives of naphthalene and phenanthrene, phenanthrene compounds clearly dominated (Figure 10).

**Figure 11:**
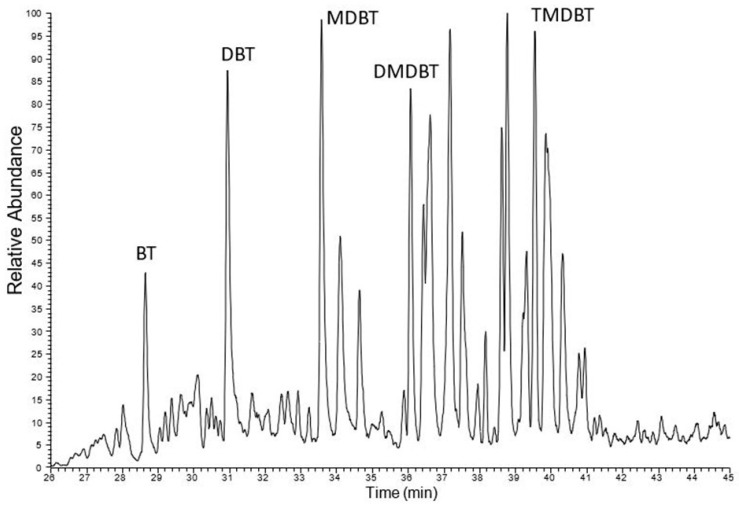
Sulfur compounds derived from benzothiophene in a sample of copper shale from LGOM (sample Jm16-1). Peak descriptions: BT—benzotiophene, DBT—dibenzotiophene, MDBT—methyldibenzotiophenes, DMDBT—dimethyldibenzotiophenes, TMDBT—trimethyldibenzotiophenes.

An analysis of the sulfur compound composition across all studied samples reveals notable differences, particularly in the content of dibenzothiophene and dimethyldibenzothiophenes. Another group of sulfur compounds—benzonaphthothiophenes—was also examined. These compounds were previously studied in Kupferschiefer from KGHM mines [51], where the presence of naphthothiophene and its alkyl derivatives was confirmed in all shale samples. In the currently analyzed samples, all benzonaphthothiophene derivatives were present in the samples from LGOM, suggesting that their occurrence is characteristic of copper mineralization (Figure 11). Among the borehole samples, the full suite of benzonaphthothiophene compounds was identified only in borehole P (sample P-2), while in borehole R, only benzonaphthothiophene itself was detected.

The elevated sulfur content observed in bitumens can be attributed to the presence of kerogen type II-S, which is characterized by an unusually high proportion of organically bound sulfur (Figure 7). This type of kerogen typically forms under euxinic depositional conditions, where limited oxygen and abundant sulfate facilitate sulfur incorporation into organic matter. During thermal maturation, kerogen II-S releases sulfur-rich compounds into the bitumen fraction, including thiophenes, sulfides, and other organosulfur species. As a result, bitumens derived from such source rocks often exhibit significantly higher sulfur concentrations. The presence of kerogen II-S not only explains the geochemical signature of the bitumens but also provides insight into the paleoenvironmental conditions during sediment deposition. An example is the presence of sulfur compounds of the methyldibezothiophene type, which are present in most samples, especially Jm16-1 (Figure 11).

### 2.8. Results of TG/DTG/FTIR Analysis

**Figure 12:**
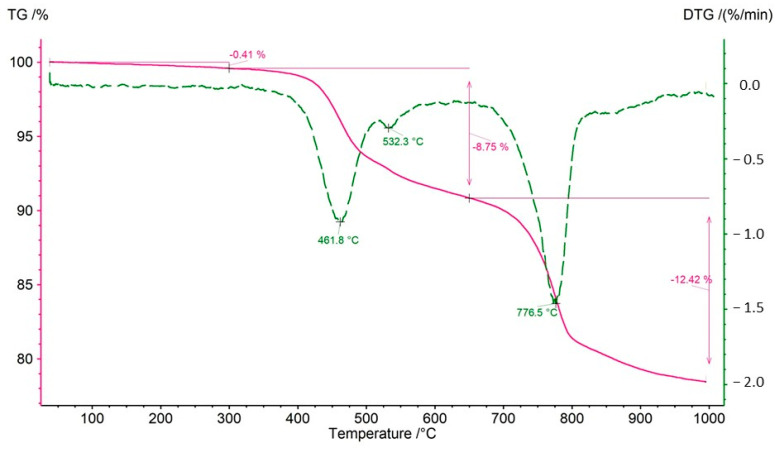
TG and DTG curves obtained for sample CG-4. The mass loss with a maximum at 532 °C results from the decomposition of covellite.

Both bulk rock samples and isolated kerogen fractions were subjected to analysis. During the thermal decomposition of the rock samples, relatively minor weight losses were observed in the temperature range of 40–300 °C, indicating a limited presence of clay minerals undergoing dehydration (Table 7). The mass loss observed between 300 and 650 °C is primarily attributed to the decomposition of organic matter. However, in certain samples (CG-4 and OK-2), an additional effect on the DTG curve at approximately 535 °C suggests the decomposition of sulfide ore minerals, most likely covellite (Figure 12). In an inert atmosphere, covellite undergoes thermal dissociation according to the reaction: 2CuS → Cu_2_S + 0.5 S within the temperature range of 400–550 °C [52]. The decomposition of sulfides at 535 °C is further supported by the presence of asymmetric S=O stretching vibrations with wavenumbers of 1373 and 1348 cm^−1^. A significant mass loss (12–26%) in the analyzed samples (except for sample OK-2) in the range of 650–1000 °C indicates the presence of carbonates—most commonly calcite, and in samples Jm16-1 and Ko-2, dolomite.

The thermal decomposition of kerogen samples is typically a two-stage process. The first effect occurs at approximately 230–260 °C, while the second appears at around 440–460 °C (Table 8). In the case of kerogen isolated from samples R-6, CG-4, P-2, and OK-2, a third effect is observed at temperatures exceeding 500 °C. This is attributed to the decomposition of metal sulfides that were not entirely removed during the kerogen extraction process. As described above, the presence of sulfides—most likely covellite—was identified in the whole rock samples CG-4 and OK-2. Gas analysis of the volatiles released during kerogen pyrolysis supports this hypothesis. The total mass loss during the pyrolysis of kerogen samples is relatively low, ranging from 22.40% to 41.21% (Table 8).

**Figure 13:**
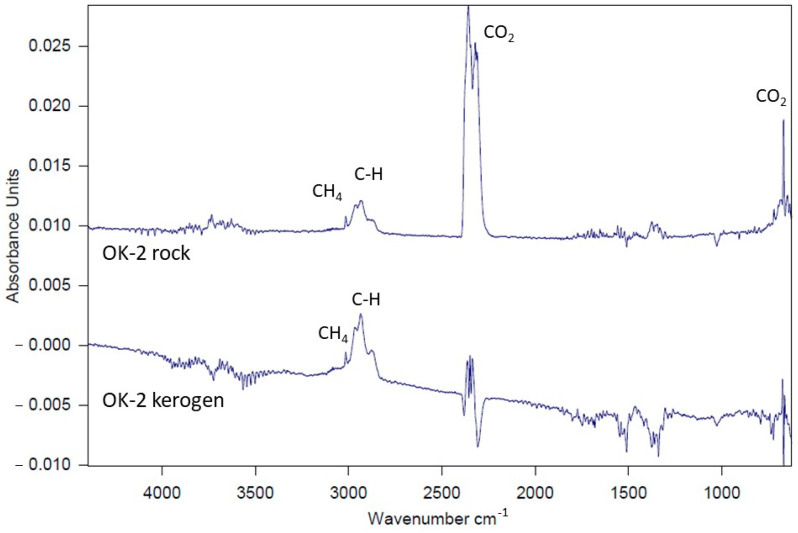
Comparison of spectra obtained at 450 °C for bulk rock samples and separated kerogen from the OK-2 sample.

In the first (I) stage of kerogen decomposition, at approximately 250 °C, FTIR spectra typically reveal the release of CO_2_. An exception is observed in the kerogen from sample Jm16-1, which shows S=O stretching vibrations; and in the kerogen from sample R-6, where stretching vibrations corresponding to C=C and C–O bonds are detected at this temperature. At 450 °C (Stage II), emission spectra characteristic of the main pyrolysis phase of kerogen are recorded. Most commonly, vibrations corresponding to the following functional groups are observed: CO_2_, CH_4_, and C–H. In some samples, vibrations from C=C or S=O groups also appear. A comparison of gas emissions between whole rock samples and corresponding kerogen samples at this temperature reveals that the FTIR spectra of kerogen decomposition are generally more intense (Figure 13—comparison for sample OK-2), although the qualitative profiles of both sample types remain comparable.

**Figure 14:**
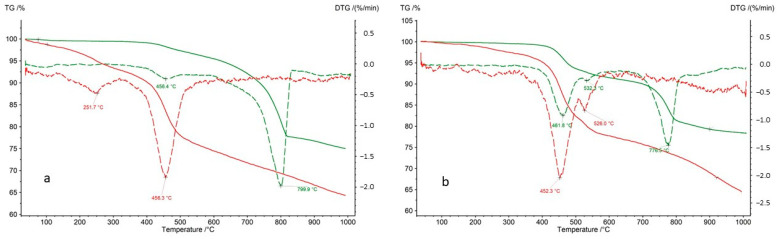
TG/DTG curves for bulk rock (green lines) and their corresponding kerogen (red lines) for samples: (**a**)—Jm02-1 and (**b**)—OK-2.

As mentioned above, TG–DTG analysis of kerogen decomposition reveals mass losses at approximately 250 °C (Stage I of decomposition). This stage is completely undetectable on the TG curves of whole rock samples, as illustrated in Figure 14a,b. The figures below present TG curves (solid lines) and DTG curves (dashed lines) for individual samples, with results for the rock sample shown in green and for the corresponding kerogen sample in red, plotted on the same graph. According to FTIR gas analysis (S=O bond vibrations), the mass loss observed at around 250 °C results from the decomposition of organic compounds containing sulfur in the form of sulphones. This effect is masked in the whole rock samples due to the presence of other inorganic components.

In the case of kerogen separated from samples R-6, CG-4, and OK-2, peaks at temperatures above 500 °C are clearly visible, originating from the decomposition of metal sulfides (example Figure 14b—sample OK-2).

### 2.9. Determination of the Kinetics of Organic Matter Decomposition

Kinetic parameters of the reaction were determined based on TG curves obtained from thermal experiments conducted at different heating rates. The temperature range considered for the calculations was 200–600 °C. The isoconversional Kissinger–Akahira–Sunose (KAS) method was applied, which employs the following kinetic equation [53]:

(2) $$\ln\left(\frac{\beta }{T^{2}}\right)=ln\frac{AR}{E_{a}g(\alpha )}-\frac{E_{a}}{RT}$$

where *A*—Arrhenius coefficient (s^−1^), *E_a_*—activation energy [kJ mol^−1^], *R*—gas constant (8.3145 kJ/mol K^−1^), *T*—temperature (K), *t*—time (min), *α*—the degree of conversion, expressed as the ratio of mass loss at a given temperature to the total mass loss within the considered temperature range. The conversion degree α represents the amount of material pyrolyzed at a given temperature.

**Figure 15:**
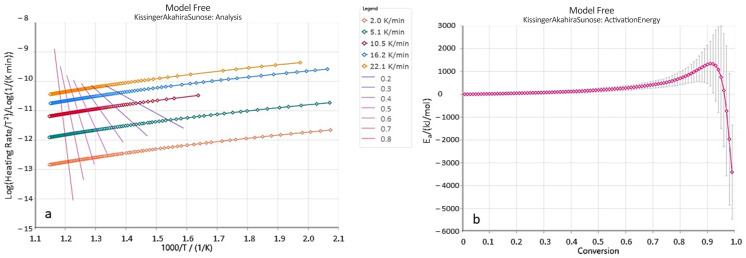
KAS (Kissinger-Akahira-Sunose) analysis for sample B-3: (**a**)—linear fit curves are shown for each conversion (conversion levels increase from right to left on the graph), (**b**)—the dependence of activation energy on the degree of conversion α.

Figure 15a presents the relationship between Log(β/T^2^) and the inverse temperature (1/T) for data points corresponding to the same conversion degree, for sample B-3. Straight lines are shown for conversion degrees ranging from 0.2 to 0.8. The slopes of these lines were used to calculate the activation energy values for the corresponding conversion degrees α (Figure 15b). As illustrated in the plots in Figure 15a, the isoconversional lines within the considered conversion range are not parallel, and their slopes vary with the progress of the pyrolysis process. This indicates a change in the reaction rate—and consequently, in the kinetic parameters—during pyrolysis. However, the values of activation energy and the Arrhenius pre-exponential factor (A_0_) generally remain relatively constant up to a conversion degree of approximately 0.7 or 0.8. Therefore, Table 9 presents the average values of kinetic parameters for conversion degrees α in the range of 0.2 to 0.8.

As shown (Table 9), the obtained average activation energy values are relatively high, ranging from 107.11 to 341.31 kJ/mol. The variability of the kinetic parameters for hydrocarbon decomposition reactions is commonly attributed to differences in the type of organic matter present [54]. Peters et al. [55], on the other hand, highlight the influence of variations in the mineral composition of source rocks. It should be noted that the overall decomposition process of the rock involves not only kerogen cracking but also the breakdown of other inorganic components within the sample. These are often interconnected and overlapping reactions [56,57]. The samples of copper-bearing shales studied here exhibit differing activation energies, which can be attributed to variations in both the organic matter composition and the mineral constituents. Therefore, the kinetic parameters derived from TG curves of whole rock decomposition represent the combined effect of all concurrent reactions, and the measured activation energy reflects an overall (apparent) activation energy [58]. As demonstrated by basic TG/DTG analysis and Rock-Eval data (particularly the MINC value—Mineral Inorganic Carbon—Table 1), most of the analyzed samples contain significant amounts of carbonate minerals, namely calcite and dolomite, which contribute to the elevated activation energy. The analyzed samples show considerable heterogeneity in their geochemical and mineralogical properties, including differences in total organic carbon (TOC) content, the abundance of clays and carbonates, and the type and thermal maturity of the organic matter. These factors are known to influence the thermal decomposition behavior of shales. Higher TOC content and the presence of more hydrogen-rich kerogen fractions tend to lower the apparent activation energy, while more inert organic matter (e.g., type III kerogen) or strong association with mineral surfaces (particularly clays and carbonates) can lead to higher apparent activation energies due to physical protection or catalytic effects.

## 3. Discussion

Black shales, referred to as copper-bearing shales (Kupferschiefer), represent the richest horizon of the Cu–Ag ore deposit exploited within the Fore-Sudetic Monocline. The mineralized zone is located within transgressive formations deposited in a subtidal or reducing environment, predominantly consisting of black shales (less commonly dark gray sandstones and carbonate rocks), which occur between red, continental-origin layers and evaporites [59]. The copper-bearing shale horizon of the Lower Zechstein in Central Europe, containing on average 4% organic matter, was formed in a stagnant basin under anoxic conditions with the involvement of sulfate-reducing bacteria. Numerous researchers have reported a positive correlation between the degree of ore mineralization and the organic matter content. The relatively shallow depth of the marine basin promoted higher organic productivity [24]. The organic matter content (TOC) determined in this study varies, reaching up to 15.89% (in the case of sample CG-4). Similar high TOC values exceeding 15% were also reported by Kotarba et al. [25] and Pieczonka et al. [31].

The issue of the origin of natural gas in the Fore-Sudetic Monocline has been addressed in numerous scientific studies [25,36]. These works have established that the copper-bearing shale (Kupferschiefer) exploited by KGHM Polska Miedź S.A. contains a significant amount of type II or II/III organic matter with a variable degree of thermal maturity [28,37], and exhibits high hydrocarbon generation potential [35]. The dominant component is type II kerogen—autochthonous sapropelic material derived primarily from marine aquatic organic matter [8,9,19,23,25,38,60]. This material, mainly associated with the sedimentation process of the copper-bearing shale, was most likely deposited under reducing environmental conditions [51], with its sources including algae, bacteria, and phytoplankton, along with the products of their decomposition [38,60]. In contrast, type III kerogen—terrestrial organic matter—constituted a significantly smaller proportion [30,31,51,60].

Sun and Püttmann [29] analyzed 37 samples of copper-bearing shale to determine the role of kerogen in the enrichment of the shale rock with ore minerals. The authors identified two distinct types of kerogen within the examined samples. One type had undergone only thermal alteration, while the other had also experienced post-sedimentary oxidation. The hydrogen index (HI) was significantly lower in the oxidized samples, whereas the oxygen index (OI) increased in comparison to the non-oxidized samples. With increasing oxidation, a transition of kerogen type from II to III was observed, which is also supported by findings of Kotarba et al. [25]. The presence of pyrobitumens and the decrease in the HI may suggest that hydrogen was extracted from the kerogen during the process of thermochemical sulfate reduction (TSR).

The research results presented in this article indicate the presence of type II and type III/II kerogen. A significant proportion of the analyzed Kupferschiefer samples are characterized by the presence of transitional type III/II kerogen. Based on previous studies [61], it was found that the comparison of FTIR spectra of gases released at 450 °C facilitates and refines the interpretation of the kerogen type present in a sample. These studies revealed the following patterns: Type II kerogen is characterized by a relatively weak methane peak, intense CO_2_ release, and often the presence of C=C stretching vibrations. Type III kerogen typically does not exhibit a methane (CH_4_) peak. In most cases, C=C vibrations are also absent. Additionally, CO_2_ peaks are generally of low intensity. Based on these criteria, the obtained results are summarized in Table 10.

As demonstrated, the results of TG-FTIR analysis often corroborate the kerogen type identified by RE analysis. In many cases where the RE results are ambiguous—indicating a transitional type III/II—TG-FTIR analysis reveals a predominance of one of the two types (II or III). The maturity of the organic matter, expressed by the T_max_ parameter, falls within the oil window for most of the analyzed samples. This is consistent with the findings of Pedersen et al. [33] for Kupferschiefer from the NW European Northern Permian Basin. Similar results were obtained through the analysis of Py-GC/FID chromatograms, confirming the oil–gas potential of the organic matter contained in the studied copper-bearing shales.

Attention should also be paid to the presence of sulfur compounds in some FTIR spectra. We note that the samples showing stronger absorption bands associated with organic sulfur functional groups in the FTIR spectra are indeed the ones that also exhibit elevated sulfur contents in the elemental analyses (e.g., sample Jm16-1). This agreement supports the interpretation that the observed FTIR sulfur signals reflect organically bound sulfur rather than contributions from inorganic sulfides or sulfates. In addition, these sulfur-rich samples also display higher hydrogen indices in the Rock-Eval data, which is consistent with the presence of type II-S kerogen.

Additionally, genetic and isotopic studies based on biomarker composition and Sofer diagrams [62] enabled the assessment of the type of source organic matter. According to the Sofer diagram, it can be inferred that the bitumen extracts were most likely generated from organic matter deposited in a marine environment. A similar isotopic correlation between saturated and aromatic hydrocarbons was presented by Kotarba et al. [25], who demonstrated the dominance of algal type II kerogen over the mixed type II/III kerogen.

**Figure 16:**
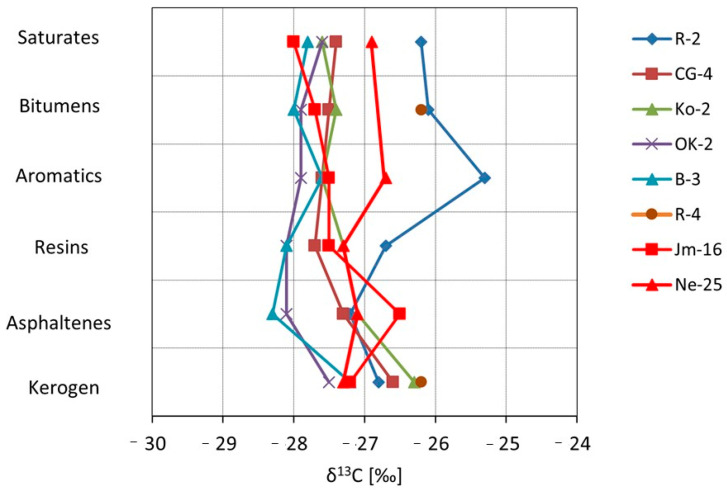
Isotope correlation curves for bitumen extract samples, their fractions and kerogen.

**Figure 17:**
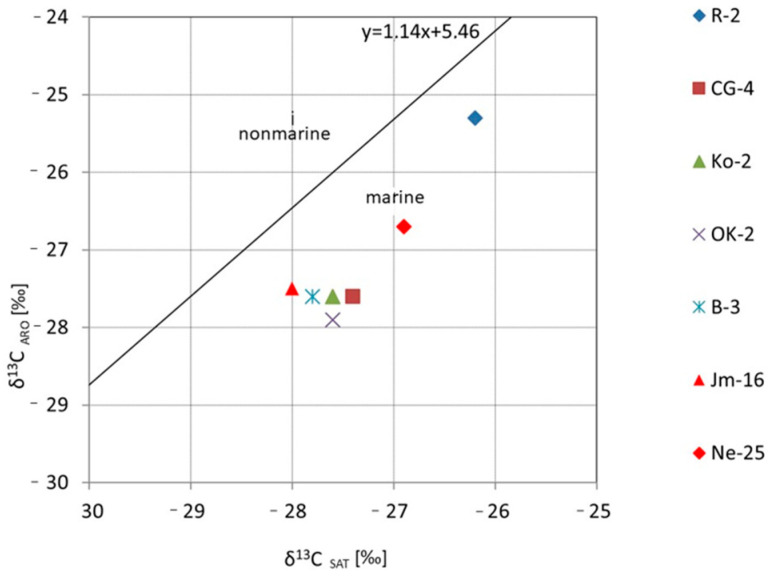
Graph showing the origin of organic matter for copper shale samples (based on Sofer, [62]).

The isotopic correlation curves were used to illustrate the obtained results (Figure 16). Sample R-4 stands out distinctly from the others in terms of its isotopic composition, with δ^13^C values of bitumens as well as the saturated and aromatic fractions shifted toward higher values. This may result from a different, more oxidized facies, in which organic compounds rich in ^12^C are preferentially released during the oxidation process, leaving the residual organic matter enriched in ^13^C. The observed isotopic trends and the correlation between the carbon isotopic composition of asphaltenes and kerogen (Figure 17) indicate that the bitumens are syngenetic with their corresponding kerogen

The hydrogen contents found in Kupferschiefer are not high and cannot be of industrial significance. However, considering the fact that elevated hydrogen and other gas contents were generally found in the lowermost layers of the copper ore deposit, i.e., in samples from both the sandstone and copper-bearing shale series [26], one should lean toward a genesis resulting from gas migration from sub-Permian formations. Bojarski et al. [63] indicate that the migration pathway for gases generated in the lower layers (probably Carboniferous) is the sub-Permian unconformity surface, constituting a contact zone with the overlying reservoir rocks. Along this zone, gases can move in various directions, with the vertical migration path considered the most favored, which could have run through, among others, Variscan and Laramide dislocations, as well as cores and wings of anticlines exposed by post-Variscan erosion. A barrier to further gas migration may be the poorly permeable Zechstein evaporites: anhydrites and the oldest rock salt [63,64], found at the top of copper ore deposits, thus serving a sealing function. However, the presence of hydrogen requires further research, particularly understanding the mechanisms of hydrogen release from organic matter, regardless of the quality of the generation potential and thermal conditions.

## 4. Materials and Methods

### 4.1. Rock Eval Pyrolysis

Pyrolytic analyses were conducted using a Rock-Eval 6 Turbo instrument (Vinci Technologies, Nanteree, France). Rock-Eval analysis is a widely used method for evaluating source rocks through open-system pyrolysis. Sample preparation involved crushing the rock material to a grain size below 0.2 mm. In the first stage of the analysis, the sample was pyrolyzed in a pyrolysis oven under a nitrogen atmosphere, using a programmed temperature ramp from 300 to 650 °C at a rate of 25 °C/min. The thermal decomposition products—hydrocarbons (HC), carbon dioxide (CO_2_), and carbon monoxide (CO)—were quantified using appropriate detectors. In the second stage, the sample was combusted in an oxidation oven under an air atmosphere, with a programmed temperature ramp from 300 to 850 °C at 20 °C/min. The combustion products—CO_2_ and CO—were quantitatively determined using an infrared (IR) detector.

The analysis yields the following parameters: S1 [mg HC/g rock]—quantity of free hydrocarbons present in the sample and released at 300 °C during pyrolysis; S2 [mg HC/g rock]—amount of hydrocarbons generated during primary kerogen cracking at 300–650 °C; S3 CO [mg CO/g rock]—CO from organic origin; Tmax—temperature at the S2 peak maximum, indicating the point of maximum hydrocarbon generation. Based on these measured values, the following indices are calculated: PI (Production Index) = S1/(S1 + S2); HI (Hydrogen Index) = S2/TOC [mg hydrocarbons per g TOC]; OI (Oxygen Index) = S3/TOC [mg CO_2_ per g TOC]; PC (Pyrolytic Carbon)—total carbon content from products generated during pyrolytic thermal degradation [%]; RC (Residual Carbon)—total carbon content from products generated during oxidative thermal degradation [%]; TOC (Total Organic Carbon)—total organic carbon content calculated as the sum of carbon contained in free hydrocarbons (S1), hydrocarbons generated from kerogen cracking (S2), and CO and CO_2_ from combustion of organic carbon [%]; MIN C (Mineral Carbon)—total mineral carbon content calculated as the sum of carbon contained in decomposition products from carbonate minerals in both pyrolysis and oxidation stages [%].

### 4.2. Gas Chromatography (Py-GC/FID)

The study of the high-temperature pyrolysis process coupled with capillary gas chromatography (Py-GC/FID) was conducted using a multi-shot pyrolyzer (Multi-Shot Pyrolyzer EGA/PY-3030D) from Frontier Laboratories LTD (Koriyama, Japan), connected to a gas chromatograph (GC-2010 Plus) equipped with a flame ionization detector (FID) from Shimadzu (Kyoto, Japan) [65].

Samples weighing approximately 5–10 mg were pyrolyzed in an inert helium atmosphere at a programmed temperature of 500 °C for a duration of 0.4 min. During the pyrolysis process, the interface temperature was maintained at 250 °C, and additional equipment was used, including a MicroJet Cryo-Trap (MJT-1030 Ex, Quantum Analytics, Woodland, TX, USA). The thermal degradation products of the analyzed samples were identified qualitatively using a gas chromatograph equipped with an FID [66].

For chromatographic analysis, an Ultra Alloy-5 capillary column was employed, with a length of 30.0 m, an internal diameter of 0.25 mm, and a film thickness of 0.25 µm, connected to the FID. Helium (5.0, EC: 231-169-5, from SIAD Poland, Ruda Śląska, Poland) was used as the carrier gas at a constant flow rate of 1.98 mL/min. A column temperature gradient was applied, starting at 30 °C (held for 5 min) and increasing to 360 °C (held for 2 min) at a rate of 10 °C/min. The injector temperature was set at 250 °C, with a split ratio of 10:1, and the FID temperature was set at 360 °C [65,67].

The analyzed rock samples, representing the Kupferschiefer horizon, were subjected to experimental high-temperature micro-pyrolysis coupled with capillary gas chromatography (Py-GC/FID) and studied with regard to the variability of three hydrocarbon groups: light (C1–C9), liquid (C10–C15), and heavy fractions (above C15+). For each sample, a single-stage pyrolysis was applied, conducted at a programmed temperature of 500 °C for 0.4 min. The obtained results were interpreted comprehensively and allowed for the characterization of the generated products based on the hydrocarbon distribution derived from the pyrolysis of the organic matter contained in the rock. To assess the generative potential of the studied copper-bearing shales, both the percentage contribution of individual hydrocarbon fractions and a dimensionless yield index (calculated from the Py-GC/FID analysis based on peak area and sample weight) were used.

The application of the MicroJet Cryo-Trap (MJT-1030 Ex) enabled the tracking of individual thermally generated hydrocarbons (at 500 °C) released during different stages of the pyrolysis process. Additionally, the flame ionization detector (FID) coupled with the gas chromatograph allowed for the analysis and monitoring of volatile organic compounds throughout the programmed temperature range.

### 4.3. Measurement of Occluded Gases

Gas content measurement is a two-stage process involving the extraction of desorbed gas and residual gas from rock cores. Desorbed gas is the portion of the gas present in the sample’s pore space and free to migrate there. Residual gas, on the other hand, is released from the rock upon fragmentation in a confined space [68]. To obtain comparable results for samples from drill cores and from the LGOM copper mine, residual gas (also known as occluded gas) was considered in further considerations, as it was possible to determine it for all samples.

A method and equipment for measuring gas content in rocks, categorized into free and occluded gas, were developed at the Oil and Gas Institute—National Research Institute (Cracow, Poland). Rock core degassing was performed in an 8000M Mixer/Mill ball mill (SPEX SamplePrep LLC, Metuchen, NJ, USA). After degassing, the released residual gas was collected using a syringe and subsequently subjected to chromatographic analysis. The molecular composition of the gas was determined using a chromatographic analysis method using a dual-channel, valve-type AGILENT 7890 A gas chromatograph (Shanghai, China) with ChemStation (rev. B.04.03) software and a column and detector system.

### 4.4. Extraction of Bituminous Substance

To determine the concentration of bitumen, an extraction method was employed as a preparatory step for further analyses, including isotopic studies, biomarker characterization, and elemental analysis. Rock extraction was carried out using a mixture of reagent grade dichloromethane (pure p.a. CAS:75-09-2 from POCH, Avantor Performance Materials Poland, Gliwice, Poland) and reagent-grade methanol (pure p.a. CAS:67-56-1 from PureLand, Stargard, Poland) in a volumetric ratio of 93:7, with use of Soxhlet apparatus. Approximately 50 g of each sample were placed in labeled paper thimbles (32 mm in diameter and 120 mm in height) made of filter paper pre-extracted with dichloromethane for 7 h. The extraction process was continued for a total of 72 h. If rock particles were observed in the collection flask, the solution was filtered through a filter paper. The obtained bitumen solution was concentrated by evaporating the excess solvent and quantitatively transferred to a pre-weighed vessel. Once a constant mass was achieved, the yield of the extract was determined.

The obtained bitumen extract from six selected samples (Jm16-1, B-3, R-4, CG-4, Ko-2, and OK-2) was separated into four fractions using: methanol (pure p.a., CAS:67-56-1 from PureLand) dichloromethane (pure p.a., CAS:75-09-2 from POCH, Avantor Performance Materials Poland, Gliwice, Poland), toluene 99.5% (pure p.a.-basic, CAS:108-88-3 from POCH), n-hexane 99% (pure p.a.-basic, CAS:110-54-3) which were subsequently subjected to detailed isotopic analyses.

### 4.5. Isotopic Composition Analysis

Isotopic composition measurements were conducted using a Delta V Advantage isotope ratio mass spectrometer (IRMS) from Thermo Scientific (Bremen, Germany). Gas mixtures were separated using a Trace GC Ultra gas chromatograph (Bremen, Germany) equipped with a HP-PLOT/Q capillary column, 30 m in length and 0.32 mm in internal diameter. The injector temperature was set to 150 °C. A column temperature program was applied, starting at 25 °C (held for 4 min), followed by a temperature increase to 210 °C (held for 5 min). The gas components, successively separated on the column, were combusted in the reactors of the GC IsoLink (Isodat ver. 3.0) interface at 1000 °C and subsequently introduced into the IRMS (Delta V Advantage, Thermo Scientific, Bremen, Germany) for isotopic analysis.

### 4.6. Kerogen Analysis

Kerogen was isolated from rock samples by acid digestion using a mixture of hydrochloric and hydrofluoric acids, following the prior removal of bitumen and humic acids. The first step in kerogen isolation involved the removal of carbonates. For this purpose, approximately 10 g of rock sample (crushed to a grain size of 0.1–0.8 mm) were heated with a 10% HCl (Hydrochloric acid 35–38% pure p.a., CAS: 7647-01-0 from Chempur, Piekary Śląskie, Poland) solution at 60 °C. The decarbonated sample was then transferred to a Teflon reactor. The reactor was filled with a mixture of 18% HCl and 40% HF (Hydrofluoric acid 40% pure p.a., CAS: 7664-39-3, from Chempur) in a 1:2 ratio, and the sample was digested at 60 °C for 8 h. After filtering off the acids and rinsing with hot distilled water, the reactor was refilled with the same acid mixture (18% HCl and 40% HF, 1:2 ratio) and heated again for 8 h.

Following the second acid digestion, the reactor was filled with an 18% HCl solution and heated at 60 °C for another 8 h. The final stage involved four rinses with hot distilled water until the filtrate reached a neutral pH. The resulting kerogen was further purified from mineral residues using a heavy liquid (a mixture of CdJ_2_—Cadmium Iodide,99.0%, purity: 99%, CAS: 7790-80-9, from Angene (London, Great Britain), and KJ—Potassium iodide CAS: 7681-11-0, from Sigma-Aldrich (Darmstadt, Germany), with a specific gravity of 2.2 g/cm^3^). The kerogen–heavy liquid mixture was centrifuged for 20 min at 4000 rpm. The isolated kerogen was then washed with 40 mL of 18% HCl, followed by four rinses with hot distilled water until a neutral pH was achieved. Finally, the kerogen was dried under a nitrogen atmosphere in an oven at 60 °C for approximately 3 h.

### 4.7. Elemental Composition Determination

Elemental composition analysis of carbon, hydrogen, nitrogen, sulfur, and oxygen in the sample was performed using a FlashSmart 2000 elemental analyzer (Thermo Scientific, Bremen, Germany). For the analysis, 1–2 mg of the sample was weighed using a microbalance and placed in a tin or silver capsule (for oxygen determination), which was then introduced into the combustion chamber of the analyzer. In the elemental analyzer, the sample undergoes catalytic complete combustion in an oxygen (purity 5.0, EC: 231-956-9, from SIAD Poland, Ruda Śląska, Poland) atmosphere at a temperature of 950 °C (with a brief temperature spike up to 1800 °C due to the combustion of the tin capsule). The combustion products pass through a copper layer that removes excess oxygen and reduces nitrogen oxides to elemental nitrogen. The combustion gases (N_2_, CO_2_, H_2_O, SO_2_) are subsequently separated chromatographically on a 2 m long, 6 × 5 mm column filled with 5Å molecular sieve, maintained at a constant temperature of 65 °C. The concentration of each component is determined using a thermal conductivity detector (TCD), which records changes in the thermal conductivity of the carrier gas (helium of purity 5.0, EC: 231-169-5, from SIAD Poland) and uses this data to calculate the concentration of each element.

The detector signal is transmitted to a control and data acquisition computer equipped with the Eager Smart integration software (version 1.0). Quantitative calculations of the elemental composition (C, H, N, S, O) were performed using absolute calibration, by comparing the peak areas of the analyzed sample to those of a reference standard (BBOT OAS from Elemental Microanalysis, Certificate No 414626, Okehampton, Devon, UK), employing the so-called K-factor method, with data processed using the Eager Smart software (ver. 1.0, Bremen, Germany).

### 4.8. Biomarker Determination

The genetic characterization of the bituminous matter from the copper-bearing shale (Kupferschiefer) was carried out based on the distribution of n-alkanes and isoprenoids, hopane compounds, and aromatic fraction compounds, including sulfur-containing compounds, as determined by GC-FID and GC-MS analyses. Biomarker analyses of the aromatic and saturated fractions were performed using a POLARIS Q ion trap mass spectrometer equipped with an RTX-5 MS column (30 m × 0.25 mm, film thickness 0.25 µm). Helium (purity 5.0, EC: 231-169-5, from SIAD Poland) was used as the carrier gas. The temperature program applied was as follows: initial temperature of 60 °C (isothermal hold for 1 min), ramped at 4 °C/min to 310 °C, and final temperature of 310 °C (isothermal hold for 15 min). For each analysis, 1 µL of sample dissolved in n-hexane was injected. Mass spectra of the aromatic and saturated fractions were acquired in both full scan mode (TIC) and selected ion monitoring mode (SIM). The data were processed using computer software, and mass spectra of specific biomarker classes were selected for identification: hopanes (*m*/*z* = 191), steranes (*m*/*z* = 217), naphthalene derivatives (*m*/*z* = 142, 156, 170), phenanthrene compounds (phenanthrene and its methylated derivatives, *m*/*z* = 178, 192), sulfur-containing compounds (methyl dibenzothiophenes, *m*/*z* = 198), and triaromatic steroids (*m*/*z* = 231).

### 4.9. Thermal Analysis TG/DTG/FTIR

Thermal analysis was performed using a NETZSCH STA 449 F3 Jupiter^®^ analyzer (Netzsch, Selb, Germany). Powdered rock samples (~20 mg) were placed in ceramic crucibles (Al_2_O_3_), while samples of isolated kerogen were weighed in amounts of approximately 5 mg. Measurements were carried out during heating from 40 °C to 1030 °C at a constant rate of 10 °C/min. A dynamic flow of inert gas (nitrogen) was maintained throughout the entire temperature range at a flow rate of 250 mL/min.

Fourier-transform infrared spectroscopy coupled with thermogravimetric analysis (TG-FTIR) was employed to investigate the gases evolved during the thermal experiments. The measurement device—a Bruker (Poznań, Poland) “Alpha” FTIR spectrometer—was directly coupled to the STA F3 system. In this configuration, the FTIR spectrometer is mounted directly on the STA furnace, eliminating the need for any transfer lines. Spectra were recorded every 16 s over the range of 400–4000 cm^−1^, with a resolution of 4 cm^−1^. The gas cell was maintained at a constant temperature of 200 °C to prevent condensation of the evolved gases. The results were analyzed using Bruker’s dedicated OPUS software (ver. 8.5).

The specific wavenumber assignments for the main absorption bands observed in the FTIR spectra are characteristic for sedimentary organic matter and kerogen, and include, for example, the following:

- aliphatic C–H stretching at ~2920 and 2850 cm^−1^,
- aromatic C=C stretching at ~1600 cm^−1^,
- carbonyl (C=O) stretching near 1700 cm^−1^,
- C–O stretching at ~1030–1150 cm^−1^,
- and sulfur-related S=O or C–S bands at ~1050–1150 cm^−1^ and ~600–700 cm^−1^.

### 4.10. Determination of Pyrolysis Kinetics

The decomposition kinetics of copper-bearing shale samples were determined based on thermogravimetric (TG) experiments conducted on approximately 5 mg of sample, using heating rates of 2, 5, 10, 15, and 20 °C/min under an inert gas (nitrogen) flow of 50 mL/min. Prior to the experiments, rock samples were ground and sieved to a particle size below 0.08 mm to minimize the influence of heat and mass transfer limitations on kinetic parameters. The tests were performed under non-isothermal conditions within the temperature range of 40–600 °C.

The NETZSCH Kinetics Neo^®^ software (version 2.4.6.8) was used to analyze the thermochemical process data. The isoconversional integral model-free Kissinger–Akahira–Sunose (KAS) method was applied for the calculations. This method was selected based on previous experimental studies on source rocks [69].

## 5. Conclusions

Within the conducted research the selected samples of Kupferschiefer and isolated kerogen were subjected to detailed analysis. The samples originated from a mining area currently exploited for Cu-Ag ores in Poland, as well as from exploratory boreholes located in the Fore-Sudetic Monocline and northern Poland (samples from borehole R). Based on the conducted investigations, the following conclusions can be drawn:

1. The analyzed samples of copper-bearing shale can be classified as excellent source rocks for hydrocarbon generation. The maturity level of the organic matter, expressed by the Tmax parameter, for the majority of samples falls within the oil window, indicating that hydrocarbon generation processes have been initiated.
2. The results of isotopic and pyrolytic analyses (Rock-Eval and Py-GC) confirm that natural gas in the studied area may originate, at least partially, from the organic matter present in the Kupferschiefer horizon.
3. High values of the S2 parameter (up to 41.88 mg HC/g rock for samples from the LGOM copper mines, as well as up to 63.65 mg HC/g rock for samples from exploration boreholes) indicate the potential for significant hydrocarbon generation.
4. Isotopic composition trends and the correlation between the carbon isotopic composition of asphaltenes and kerogen demonstrate that the bitumens are syngenetic with their corresponding kerogen, which shows minor variability resulting from oxidation processes affecting the organic matter.
5. Thermogravimetric analysis revealed that the peak of hydrocarbon release in rock samples occurs at approximately 460 °C. In the case of isolated kerogen, an additional mass loss effect at around 250 °C is associated with the decomposition of organic compounds containing sulfur in the form of sulphones.
6. The analysis of gases released during thermal decomposition of the samples allows for a more precise determination of kerogen type. Most samples exhibit a sapropelic character, as confirmed by C/N ratios.
7. The examined copper shale samples exhibit variable activation energy values, reflecting differences in the composition of organic matter, as supported by biomarker profiles and sulfur compound content. The activation energy values suggest significant differences in the timing of hydrocarbon generation. In particular, the copper shales from the LGOM region may require a notably shorter generation time due to lower activation energy values.
8. The hydrogen content found in Kupferschiefer is rather low, not exceeding 403 μg/kg rock, and cannot be of industrial significance.

## Figures and Tables

**Table 1:** Rock-Eval parameter variability for the tested samples (parameters given in columns are characterized in the text, in Section 2.2).

Sample	Symbol on the Map	Depth	TOC	T_max_	S1	S2	S3	PI	HI	OI	EOM	SAT	ARO	RES	ASPH	Isotopes
B-3	B	1617.75	7.47	439	1.49	15.87	0.55	0.09	212	7	1795	19.7	23.1	21	36.2	−27.2
WM-1	WM	1564.70	0.11	454	0.01	0.04	0.54	0.16	36	491						
R-1	R	3106.15	0.59	441	0.17	0.29	0.23	0.37	49	39						
R-2		3106.65	3.49	459	1.13	2.71	0.19	0.30	78	5						
R-3		3106.95	1.99	457	0.56	1.60	0.15	0.26	80	8						
R-4		3107.00	7.11	457	1.95	8.02	0.20	0.20	113	3	1875	44.7	21.5	22.9	10.9	−26.8
R-5		3107.10	3.02	456	0.84	3.33	0.16	0.20	110	5	582					−26.2
R-6		3107.25	4.12	459	1.10	4.68	0.20	0.19	114	5						
R-7		3107.95	0.38	462	0.18	0.30	0.17	0.37	79	45						
CG-1	CG	2655.00	5.65	457	1.32	4.59	0.23	0.22	81	4	6533	10.1	41.7	29.9	18.3	−26.6
CG-2		2655.35	12.89	441	2.23	51.18	0.21	0.04	397	2						
CG-3		2655.50	11.65	441	1.99	50.41	0.19	0.04	433	2						
CG-4		2655.75	15.89	439	2.48	63.65	0.33	0.04	401	2						
P-1	P	1731.65	1.57	447	0.22	2.61	0.33	0.08	166	21						
P-2		1731.90	3.25	445	0.41	4.84	0.43	0.08	149	13	1514	11.54	17.61	22.70	48.20	−26.9
K-1	K	2466.10	4.98	520	0.04	0.83	0.35	0.04	17	7	296					
K-2		2466.20	3.58	538	0.04	0.50	0.72	0.08	14	20						
K-3		2466.30	1.73	524	0.03	0.29	0.60	0.08	17	35						
Ko-1	Ko	2301.00	2.88	441	0.58	8.46	0.11	0.06	294	4						
Ko-2		2301.75	6.08	446	1.11	19.50	0.16	0.05	321	3	1400	27.2	12.6	34.3	25.8	−26.6
Ko-3		2302.30	3.65	451	1.17	7.77	0.13	0.13	213	4						
Ko-4		2302.45	5.39	445	1.18	15.23	0.22	0.07	283	4						
Ko-5		2303.00	2.46	446	0.65	5.68	0.18	0.10	231	7						
Ko-6		2303.35	0.41	428	0.68	0.87	0.15	0.44	212	37						
OK-2	OK	2235.00	11.27	438	1.89	39.61	0.13	0.05	351	1	2425	22.1	41.1	26.5	10.3	−27.5
Jm02-1	LGOM *	-	8.46	442	1.79	36.30	0.29	0.05	429	3						
Jm16-1		-	2.09	437	0.54	5.68	0.38	0.09	272	18	1571	21.51	10.22	20.97	47.31	−27.3
Ja21-1		-	2.16	441	0.12	2.18	1.10	0.05	101	51						
Ne25-1		-	12.57	434	1.69	41.88	0.42	0.04	333	3	3063	8.59	38.42	17.9	35.08	−27.2
Ra15-1		-	3.16	438	0.26	6.05	0.78	0.04	191	25						
Ne-25-2		-	11.50	433	0.95	33.67	0.46	0.03	293	4						
Jm07-1		-	4.25	439	0.53	8.88	0.73	0.06	209	17						
Jm07-2		-	3.88	438	0.27	7.33	0.64	0.04	189	16						
Jm12-1		-	7.96	433	1.65	26.12	0.37	0.06	328	5	2918					
Jm16-2		-	9.85	438	1.85	32.09	0.47	0.05	326	5						
Jm08-1		-	3.46	438	0.61	7.48	0.57	0.08	216	16						
Jm08-2		-	5.71	437	0.83	14.34	0.39	0.05	251	7						
Jm02-2		-	10.71	436	2.15	37.20	0.31	0.05	347	3						

* In the case of samples marked with symbols Jm, Ne and Ra, the depth was not given because they were taken from mining workings. Gray background color highlight samples selected for detailed investigation.

**Table 2:** Results of Py-GC/FID pyrolysis analysis of tested samples, providing the composition of simulated hydrocarbon generation products at 500 °C.

Sample	Pyrolysis Products at 500 °C (Py-GC/FID)			
	Ratio Yield *	C_1_–C_9_ [%]	C_10_–C_15_ [%]	C_15+_ [%]
Jm02-1	49.46	64.34	23.08	12.58
Jm16-1	8.64	33.09	25.23	41.68
Ne25-1	45.52	63.82	27.07	9.11
B-3	27.02	39.74	35.45	24.81
R-4	31.91	54.90	27.60	17.50
R-6	4.61	75.47	21.33	3.20
CG-4	172.45	56.63	21.82	21.55
P-2	16.28	72.35	20.41	7.24
Ko-2	23.74	72.39	19.20	8.42
OK-2	41.62	66.11	22.74	11.15

* Ratio yield—dimensionless yield factor from Py-GC/FID analysis (calculated per 1 mg of rock sample).

**Table 3:** Carbon isotopic composition in bituminous extracts and individual fractions as well as in kerogen [‰ vs. PDB].

Sample	Bituminous Extract	Saturated Fraction	Aromatic Fraction	Resin Fraction	Asphaltene Fraction	Kerogen
Jm02-1	n.d.	n.d.	n.d.	n.d.	n.d.	−27.3
Jm16-1	n.d.	−28.0	−27.5	−27.5	−26.5	−27.2
B-3	−28.0	−27.8	−27.6	−28.1	−28.3	−27.2
R-2	−26.1	−26.2	−25.3	−26.7	−27.2	−26.8
R-4	−26.2	n.d.	n.d.	n.d.	n.d.	−26.2
CG-4	−27.5	−27.4	−27.6	−27.7	−27.3	−26.6
Ko-2	−27.4	−27.6	−27.6	−27.3	−27.1	−26.3
OK-2	−27.9	−27.6	−27.9	−28.1	−28.1	−27.5
P-5	−27.6	n.d.	n.d.	n.d.	n.d.	−26.9

where n.d.—not determined.

**Table 4:** Results of the elemental analysis of bitumen extract.

Sample	Elemental Composition					Atomic Ratio			
	C [%]	H [%]	N [%]	S [%]	O [%]	H/C	O/C	N/C	S/C
Jm-16-1	79.38	9.72	1.13	8.69	1.08	1.47	0.01	0.012	0.041
Ne25-1	82.42	9.01	1.11	4.62	2.83	1.31	0.03	0.012	0.021
B-3	81.53	9.12	0.99	1.03	7.35	1.34	0.07	0.010	0.005
R-4	84.56	8.30	0.47	1.51	5.16	1.18	0.05	0.005	0.014
R-6	82.90	9.65	0.62	3.00	3.83	1.40	0.03	0.006	0.019
CG-4	84.48	8.97	1.06	4.21	1.28	1.27	0.01	0.011	0.05
P-2	85.02	7.83	0.67	1.73	4.75	1.11	0.06	0.010	0.02
Ko-2	86.84	9.15	0.95	2.14	0.92	1.26	0.01	0.010	0.02
OK-2	87.68	9.27	0.84	1.28	0.94	1.27	0.01	0.008	0.005

**Table 5:** Results of the elemental analysis of kerogen isolated from Kupferschiefer.

Kerogen from Sample	C_ker_ [%]	Ash [%]	Mineral Matter [%]	Elemental Composition Without Mineral Matter [%]				S [%]	Atomic Ratio				
				C	H	N	O		H/C	O/C	N/C	S/C	C/N
Jm02-1	8.40	26.86	31.92	85.39	7.22	1.97	5.09	5.28	1.01	0.04	0.003	0.023	50.604
Jm16-1	2.06	21.70	31.84	80.30	5.91	2.16	4.08	15.29	0.88	0.04	0.004	0.071	43.297
B-3	7.37	15.40	21.16	83.93	5.58	2.75	4.60	8.24	0.80	0.04	0.005	0.037	35.595
R-4	7.04	12.99	20.40	84.29	4.79	1.87	3.82	11.58	0.68	0.03	0.003	0.052	52.727
R-6	4.10	9.84	14.93	85.73	4.79	1.91	4.36	7.83	0.67	0.04	0.003	0.034	52.491
CG-4	15.73	12.57	21.19	80.91	6.52	1.91	4.03	13.85	0.97	0.04	0.003	0.064	49.391
P-2	3.19	11.42	17.07	82.62	4.97	2.92	5.91	8.62	0.72	0.05	0.005	0.039	32.961
Ko-2	6.01	19.23	29.22	81.08	5.88	1.78	3.66	15.38	0.87	0.03	0.003	0.071	53.204
OK-2	11.13	16.19	24.16	82.08	6.18	2.09	4.15	12.13	0.90	0.04	0.004	0.055	45.730

**Table 6:** Indicators calculated based on the composition of n-alkanes and isoprenoids.

Sample	CPI_(Total)_	CPI_(17–23)_	CPI_(25–31)_	Pr/Ph	Pr/nC_17_	Ph/nC_18_	n-C_max_
Jm16-1	0.95	0.96	1.02	1.00	1.15	0.69	n-C_24_
Ne25-1	0.98	0.99	1.07	1.19	1.17	0.43	n-C_18(19)_
B-3	1.00	1.03	1.01	1.12	0.54	0.30	n-C_20_
R-4	1.00	1.01	1.08	0.63	0.36	0.30	n-C_20_
CG-4	1.05	1.05	1.03	n.d.	n.d.	0.48	n-C_20_
P-2	1.00	1.03	1.01	0.99	0.71	0.15	n-C_20_
Ko-2	1.00	1.03	1.01	0.54	0.40	0.32	n-C_20_
OK-2	1.10	1.09	1.39	1.04	1.45	0.60	n-C_20_

n.d.—not determined.

**Table 7:** Mass loss [%] in particular temperature ranges [°C].

Sample	Temperature Range [°C]			Rest of the Sample Mass [%]
	40–300	300–650	650–1000	
Jm02-1	0.53	5.30	19.29	74.88
Jm16-1	0.37	3.17	24.96	71.15
Ne25-1	0.20	7.39	14.23	78.18
B-3	0.52	6.19	23.52	69.77
R-4	0.26	5.37	13.90	80.47
R-6	0.14	4.25	26.11	69.50
CG-4	0.41	8.75	12.42	78.42
P-2	0.61	4.79	17.43	77.17
Ko-2	0.11	4.20	33.07	62.62
OK-2	0.59	6.59	3.98	88.84

**Table 8:** Total mass loss and maximum mass loss temperature for kerogen samples.

Kerogen from Samples	T_TGmax_ [°C]			Total Mass Loss [%]
	I	II	III	
Jm02-1	252	455		41.21
Jm16-1	261	456		37.38
B-3	248	451		34.25
R-4	263	514		23.72
R-6	257, 278	444	514	22.40
CG-4	-	450	528	36.16
P-2	265	463	524	25.59
Ko-2	235	437		29.63
OK-2	238	459	544	30.71

**Table 9:** Average values of kinetic parameters for the tested samples of Kupferschiefer for conversion degrees from 0.2 to 0.8.

Sample	E_a_ (kJ/mol)	Log A (Log(1/s))
Jm02-1	198.87	10.68
Jm16-1	107.11	4.51
Ne25-1	154.67	4.61
B-3	238.94	13.14
R-4	341.31	19.45
R-6	242.13	13.11
CG-4	319.59 *	20.69
P-2	148.49	7.15
Ko-2	170.71	9.31
OK-2	309.92	18.53

* For sample CG-4 the complete conversion occurs for α = 0.51, which causes E_a_ to drop below 0 above this conversion. Therefore, the average was calculated for α in the range of 0.2–0.5.

**Table 10:** Comparison of kerogen types identified based on RE and TG-FTIR test results for whole rock samples.

Rock Sample	Kerogen Type Based on RE	TG-FTIR Results	
		Kerogen Type	Indicators
Jm02-1	II	II	CH_4_, CO_2_, C=C
Jm16-1	III/II	III	Lack of CH_4_, C=C, low CO_2_
Ne25-1	II	II	CH_4_, CO_2_
B-3	III/II	II	CH_4_, CO_2_, C=C
R-4	III/II	III	Lack of CH_4_, C=C, low CO_2_
R-6	III/II	III	Lack of CH_4_, C=C, low CO_2_
CG-4	II	II	CH_4_, CO_2_
P-2	II	II	CO_2_
Ko-2	III/II	II	CH_4_, CO_2_
OK-2	II	II	CH_4_, CO_2_

## Data Availability

The data presented in this study are available in data repository: “Dataset for geochemical characterisation of Kupferschiefer”, https://data.mendeley.com/datasets/6b9g6g9y5d/ accessed on 23 September 2025.

## Abbreviations

CPI	Carbon Preference Index
ARO	Aromatic compounds
ASPH	Asphaltene compounds
EOM	Extractable organic matter
HI	Hydrogen Index
LGOM	Legnica–Głogów Copper District
MIN C	Mineral Carbon
OI	Oxygen Index
PC	Pyrolytic Carbon
PDB	Pee Dee Belemnite
PI	Production Index
RC	Residual Carbon
RES	Resin compounds
SAT	Saturated Hydrocarbons
TOC	Total Organic Carbon
TSR	Thermochemical Sulfate Reduction

## Appendix A

**Table A1:** Occluded gas content in Kupferschiefer samples.

No. as in Figure 5	Sample	Gas Occluded	CH_4_	H_2_	PyGC Ratio Yield
		[μg/kg of Rock]			[-] *
1	B-3	64,374	2394	107.1	27.0
2	WM-1	36,335	106	0	0.0
3	R-1	54,542	1710	69.7	0.1
4	R-2	62,623	2130	140.2	2.8
5	R-3	82,408	15,661	157.0	0.4
6	R-4	50,599	2503	85.6	31.9
7	R-5	67,132	12,045	171.6	0.9
8	R-6	45,786	8637	0	4.6
9	R-7	48,803	193	0	0.1
10	CG-1	101,739	2505	290.3	4.9
11	CG-2	169,920	19,728	119.0	192.6
12	CG-3	280,677	139,905	144.1	49.8
13	CG-4	208,490	13,796	172.9	172.5
14	P-1	73,768	1475	0	1.0
15	P-2	90,234	10,467	207.7	16.3
16	K-1	44,683	266	84.9	0.3
17	K-2	41,157	220	115.1	0.2
18	K-3	71,755	524	67.3	0.1
19	Ko-1	30,958	1306	0	5.0
20	Ko-2	109,254	985	119.6	23.7
21	Ko-3	32,798	3586	137.5	2.8
22	Ko-4	81,890	5700	181.7	8.1
23	Ko-5	88,740	3323	121.8	1.0
24	Ko-6	49,699	3715	172.7	0.2
25	OK-2	76,608	6049	80.9	41.6
26	Jm02-1	68,205	145	158.4	49.5
27	Jm16-1	12,829	45	43.3	8.6
28	Ja21-1	60,145	265	210.6	0.7
29	Ne25-1	357,644	3093	403.1	45.5
30	Ra15-1	219,026	805	132.7	9.3
31	Ne-25-2	152,897	2444	316.2	49.0
32	Jm07-1	255,452	1396	0	6.0
33	Jm07-2	25,882	93	0	2.9
34	Jm12-1	129,068	999	0	26.9
35	Jm16-2	241,005	1023	0	42.7
36	Jm08-1	50,507	6	0	11.0
37	Jm08-2	17,173	154	0	8.7
38	Jm02-2	170,027	9064	353.1	37.0

* Ratio yield—dimensionless yield factor from Py-GC/FID analysis (calculated per 1 mg of rock sample).
